# Body Mass Index Trajectories in the First 5 Years and Associated Antenatal Factors

**DOI:** 10.3389/fped.2021.622381

**Published:** 2021-02-19

**Authors:** Molly Mattsson, Deirdre M. Murray, Colin P. Hawkes, Mairead Kiely, Carol Ní Chaoimh, Fergus P. McCarthy, Regien Biesma, Fiona Boland

**Affiliations:** ^1^Division of Population Health Sciences, Royal College of Surgeons in Ireland, Dublin, Ireland; ^2^Department of Paediatrics and Child Health, University College Cork, Cork, Ireland; ^3^Division of Endocrinology and Diabetes, The Children's Hospital of Philadelphia, Philadelphia, PA, United States; ^4^Cork Centre for Vitamin D and Nutrition Research, School of Food and Nutritional Sciences, University College Cork, Cork, Ireland; ^5^Irish Centre for Maternal and Child Health Research, Cork University Maternity Hospital, University College Cork, Cork, Ireland; ^6^University Medical Center Groningen, University of Groningen, Groningen, Netherlands; ^7^Data Science Centre, Royal College of Surgeons in Ireland, Dublin, Ireland

**Keywords:** growth, childhood obesity, epidemiology, growth mixture modeling, IGF

## Abstract

**Background:** The increasing prevalence of childhood obesity is an important public health issue and the development of obesity in early life and associated risk factors need to be better understood. The aim of this study was to identify distinct body mass index trajectories in the first 5 years of life and to examine their associations with factors identified in pregnancy, including metabolic parameters.

**Methods:** BMI measurements from 2,172 children in Ireland enrolled in the BASELINE cohort study with BMI assessments at birth, 2, 6, and 12 months, and 2 and 5 years were analyzed. Growth mixture modeling was used to identify distinct BMI trajectories, and multivariate multinomial logistic regression was used to assess the association between these trajectories and antenatal factors.

**Results:** Three distinct BMI trajectories were identified: normal (89.6%); rapid gain in the first 6 months (7.8%); and rapid BMI after 12 months (2.6%). Male sex and higher maternal age increased the likelihood of belonging to the rapid gain in the first 6 months trajectory. Raised maternal BMI at 15 weeks of pregnancy and lower cord blood IGF-2 were associated with rapid gain after 1 year.

**Conclusion:** Sex, maternal age and BMI, and IGF-2 levels were found to be associated with BMI trajectories in early childhood departing from normal growth. Further research and extended follow-up to examine the effects of childhood growth patterns are required to understand their relationship with health outcomes.

## Introduction

Obesity is a growing public health issue globally and a risk factor for multiple non-communicable diseases (NCD) (1–6). The worldwide prevalence of obesity has increased from 3.2% in 1975 to 10.8% in 2014 in men, and from 6.4 to 14.9% in women. The prevalence in the Irish population surpasses the global mean and has been estimated as 26% both men and women (7). Children and adolescents with obesity have been found to be around five times more likely to have obesity in adulthood compared to children without obesity (8). In Ireland, the prevalence of obesity at 5 years of age increased from 1.2% in 1975 to 11.3% in 2016 in boys, and from 1.2% in 1975 to 10.1% in 2016 in girls.

Tracking children's growth patterns longitudinally allows for the assessment of dynamic changes in size. This may permit a more accurate identification of young children at higher risk when compared with assessments at a single time point, and provide insight into early life determinants of childhood and adult overweight and obesity (9). Body mass index (BMI) for assessment of weight status is generally not used under the age of 2 (10) and instead the American Academy of Pediatrics recommend weight-for-length (WFL) for this age group (11). However, previous studies have shown that BMI z-scores in infancy to have a significantly higher positive predictive value for early childhood obesity than WFL-Z (12) and that raw BMI may be a better indicator of body composition at 1 months of age compared to WFL (13).

Longitudinal random-effects and latent growth curve models are commonly used to explore growth trajectories in childhood. These methods allow for individual variability; however they assess the average pattern of change and assume that individuals belong to the same underlying population, represented by a single growth curve. As an alternative, a latent class approach may be adopted, allowing researchers to identify and describe underlying subgroups within a population based on different trajectories (14). Latent class growth analysis (LCGA) estimates a mean growth curve for each class, but no individual variation around the mean growth curve is allowed. Growth mixture modeling (GMM) combines the features of the random effects model and LCGA by estimating both mean growth curves for each class and individual variation around these growth curves (15).

The development of obesogenic growth trajectories and childhood obesity is complex. From a life course model of chronic disease epidemiology perspective, childhood obesity has previously been conceptualized in a framework where individual-level factors, including biological, social, and behavioral risks, are acting within the influence of the child's family environment, which is, in turn imbedded in the context of the community environment (16). Additionally, it is helpful to consider critical periods of biological and behavioral plasticity for obesity risk, beginning as early as fetal life (17). In this study, we have aimed to investigate BMI trajectories in early life and factors identified during pregnancy. Sociodemographic, lifestyle and metabolic health factors in pregnancy, including low socioeconomic status (SES), smoking, maternal pre-pregnancy BMI, gestational weight gain (GWG), and gestational diabetes mellitus (GDM), have been consistently associated with multiple aspects of child growth, such as birthweight, growth velocity, BMI, and childhood obesity (18–21).

We previously conducted a systematic review of group-based trajectory modeling for BMI trajectories in childhood, and found that trajectories of excessive rapid gain were associated with several predictors, including high maternal pre-pregnancy BMI, GWG and smoking during pregnancy (22). The association of maternal and infant metabolic parameters, beyond pre-pregnancy BMI, GWG and GDM, in relation to offspring's longitudinal BMI trajectories does however remain largely unexplored. Insulin-like growth factor (IGF)-1 and IGF-2 are peptides primarily secreted by the liver. Small studies have previously demonstrated a correlation between cord blood IGF-I concentration and birthweight (23, 24) as well as weight at 6 months (25). Cord blood concentrations of IGF-2 have been found to be related to IGF-2 levels at age five, which in turn has been related to fat mass (24). Further, low IGF-2 levels have been documented in pre-puberal children with obesity (26). Leptin is a hormone secreted primarily by adipocytes and evidence suggests that low fetal leptin concentrations may mediate weight gain during infancy and play a role in the development of obesity (27–29). A recent study suggests that cord blood leptin may play a modest mediating role in early postnatal catch-up or catch-down in weight (30).

The aim of this study was to identify distinct BMI trajectories in the first 5 years of life using GMM methods and to examine the associations between these trajectories and factors identified during pregnancy, including maternal and fetal metabolic parameters.

## Methods

### Study Population and Design

Study subjects were participants of the Cork BASELINE (Babies after SCOPE: Evaluating the Longitudinal Impact using Neurological and Nutritional Endpoints) Birth Cohort Study (31), a mother–infant prospective birth cohort study based in Cork, Ireland. It was initiated in 2008 as a follow-up to the SCOPE (Screening for Pregnancy Endpoints) Ireland study (32), a major multi-center prospective pregnancy study involving primiparous low-risk women. Exclusion criteria included women recognized as high risk of pre-eclampsia, small for gestational age baby, or spontaneous preterm birth, as these were the primary outcomes of interest in the study. A total of 1,768 women were recruited to SCOPE Ireland at Cork University Maternity Hospital (CUMH). Each participant was interviewed by a research midwife at 14–16 and 19–21 weeks of gestation and detailed clinical information, maternal anthropometry measurements, ultrasound data, and blood specimens were collected. Umbilical cord blood was collected at birth. One thousand, five hundred thirty-seven SCOPE participants consented for their infants to participate in the BASELINE study and during a second stream of recruitment, a further 646 infants were recruited after delivery from the postnatal wards of CUMH, with a singleton pregnancy being the main inclusion criterion (31). Pediatric follow-up with in-person assessments were conducted at birth, 2, 6, and 12 months and at 2 and 5 years. Data on the child's early-life environment, diet, health, and development were recorded at each assessment. In total, 2,172 infants were followed up after birth.

### Maternal Data

Maternal age, smoking and alcohol use during pregnancy, and BMI were obtained during the SCOPE study interviews at 14–16 and 19–21 weeks, and are only available for the mothers who participated in both SCOPE and BASELINE. GDM diagnosis was established post-delivery from medical records. Education level, income, and marital status were determined at the 2 month assessment of BASELINE.

### Infant Data

Umbilical cord samples (including leptin and IGF-1 and -2 concentrations) were obtained at birth for infants in the SCOPE study and z-score variables were created using the *z-score* command in Stata. Leptin was only collected in a subset of children (*n* = 405). Gestational age (GA) at delivery was obtained from medical records, with preterm birth defined as <37 weeks gestation.

### Child Anthropometric Measurements

Measurements of child weight and length/height were obtained at birth 2, 6, and 12 months and 2 years and 5 years of age. The available BMI data at each time point is outlined in Supplementary Table 1. Naked weight was measured using digital scales at birth and at 2 months correct to the nearest 0.01 kg and at 6 and 12 months and at 2 and 5 years correct to the nearest 0.1 kg. Supine length correct to the nearest 0.1 cm was measured at birth 2, 6 and 12 months. At 2 and 5 years, standing height was measured using a wall mounted stadiometer (33). Waist circumference was measured at each assessment. All measures were performed by trained research staff.

### Statistical Analysis

BMI trajectories in the first 5 years of life were analyzed using GMM. A longitudinal change model was assumed where each growth pattern was characterized by random intercept and linear, quadratic, and cubic terms by age, allowing for curved developmental patterns. Cubic variance was fixed, thus not allowing for individual variance for cubic terms. The residual variance for BMI at 5 years was fixed to zero, as a small and not significant negative residual was identified for this variable. We used the maximum likelihood robust estimator to account for missing data by full information maximum likelihood (FIML). This process approximates missing data by estimating a likelihood function for each individual based on variables that are present, such that all the available data points are used (34). The optimal number of latent trajectories was identified based on four model-fit indices: Sample-size adjusted Bayesian information criterion (BIC), adjusted Bootstrap likelihood ratio test (BLRT), Lo-Mendell Rubin test (LMRT), entropy, and interpretability of the trajectories. A lower BIC value indicates a better model fit, while the BLRT and LMRT provide a *p*-value indicating whether a model with one less trajectory group (k-1 model) should be rejected in favor of a model with k trajectories (35). Entropy is a statistic that ranges from 0 to 1 with high values (>0.8) indicating that individuals are classified with confidence (14). Distinct trajectories were coded as a categorical variable (with k number of categories) and were named based on their visual appearance. The selected model was reproduced in children with no missing BMI data (*n* = 915).

Associations between factors identified in pregnancy and at delivery (maternal age, education level, income, marital status, smoking and alcohol use during pregnancy, BMI at 15 weeks, child sex, and cord blood leptin, IGF-1, and IGF-2) and BMI trajectories were examined using multivariate multinomial logistic regression, with the most commonly occurring trajectory chosen as the reference category. GDM and preterm delivery were not included due to their low prevalence in the study. Supplementary Figure 1 includes a flow chart for the number of children included at each stage of the study. Analysis was conducted using Mplus version 8 (36) and Stata version 14 (37). The GroLTS (Guidelines for Reporting on Latent Trajectory Studies) Checklist was used as a guide for completing analysis and manuscript preparation (38).

## Results

### BMI Trajectories in the First 5 Years

**Figure 1:**
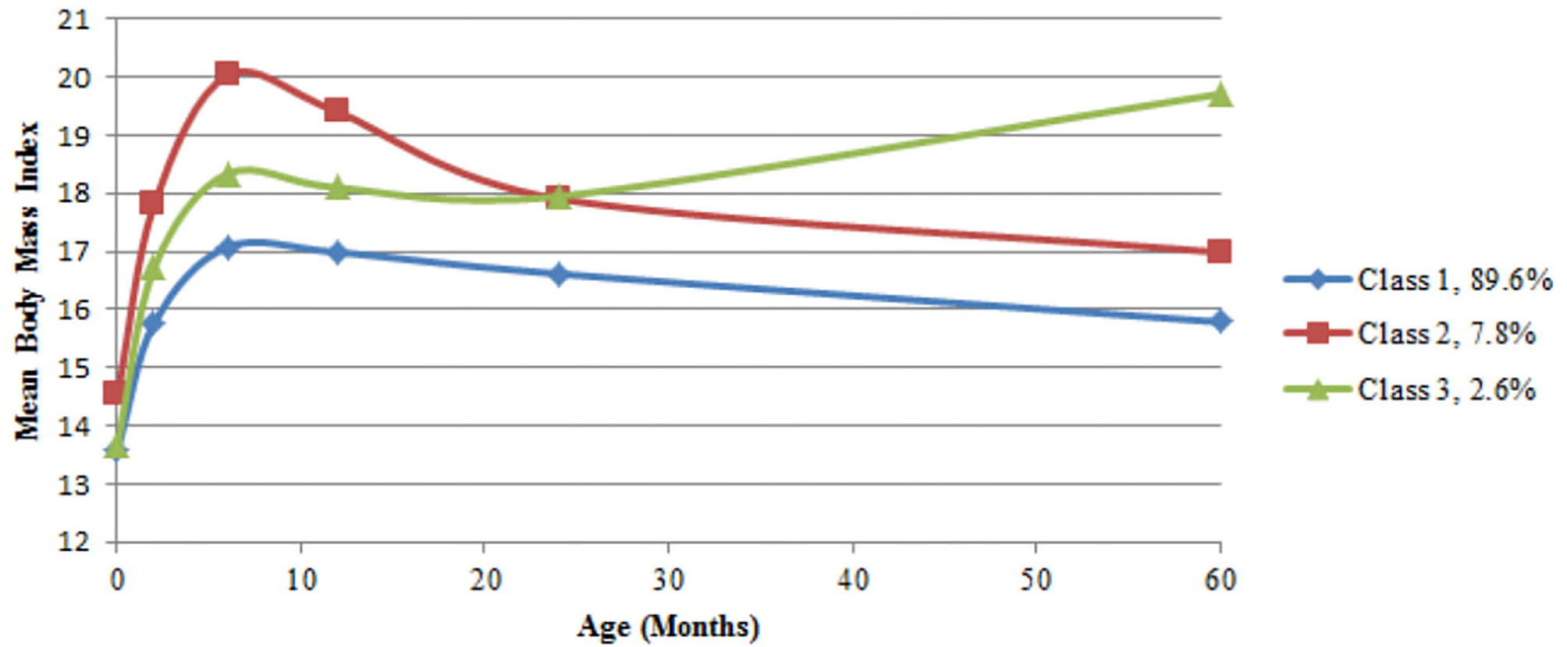
BMI trajectories. Class 1 *n* = 1,947; Class 2 *n* = 169; Class 3 *n* = 56.

Table 1 describes the distribution of demographic and clinical characteristics for the whole sample as well as according to BMI trajectories. Based on the fit indices in Table 2 the three-class model was selected. Sample-size adjusted BIC and BLRT indicated a better fit for a four trajectory model, however LMRT indicated that a four trajectory model was not significantly superior to the three trajectory one. Furthermore, the four trajectory model identified two very small subgroups (2.0 and 1.3%), which may reduce interpretability. Supplementary Table 2 outlines the average BMI and standard deviation (SD) at each time point by sex and trajectory membership. The majority of the children (89.6%, *n* = 1,947) exhibited a BMI trajectory corresponding to the 50th to 75th percentile according to WHO growth standards for both boys and girls at each time point (39). Secondly, 7.8% (*n* = 169) of children had a higher BMI at birth compared to the other two groups (75th percentile), which increased to the 95th percentile in the first 6 months, remained at 95th percentile between six and 24 months, and decreased to the 85th percentile by 5 years. Finally, 2.6% (*n* = 56) had a BMI at the 60th percentile at birth, a BMI between the 75th and 85th percentile between two and 12 months, followed by the 95th percentile at 2 years and 99th percentile at 5 years (Figure 1). Sensitivity analyses using subjects with no missing BMI data in the first 5 years (*n* = 915) showed similar trajectory patterns.

**Table 1:** Distribution of demographic and clinical characteristics according to BMI trajectory.

	**All trajectories**	**Class 1**	**Class 2**	**Class 3**	***P*-value^a^**
Average age ± standard deviation (SD), years (*n* = 1,572)	30.0 ± 4.4	30.0 ± 4.4	30.0 ± 4.5	29.7 ± 4.9	0.88
	***n*** **(%)^b^**	***n*** **(%)**	***n*** **(%)**	***n*** **(%)**	
Ethnicity (*n* = 1,950)					0.79
Irish or other Caucasian	1.911 (98.0)	1,691 (98.0)	165 (97.6)	55 (98.2)	
Other	39 (2.0)	34 (2.0)	4 (2.4)	1 (1.8)	
Educational attainment (*n* = 1,949)					0.19
No college/university education	208 (10.7)^b^	182 (10.6)	16 (9.5)	10 (17.9)	
Any college/university education	1,741 (89.3)	1,542 (89.4)	153 (90.5)	46 (82.1)	
Income level per year (*n* = 1,873)					0.23
<€43 k	515 (27.5)	451 (27.2)	44 (26.8)	20 (38.5)	
€43–63 k	436 (23.3)	380 (22.9)	42 (25.6)	14 (26.9)	
€64+	922 (49.2)	826 (49.9)	78 (47.6)	18 (34.6)	
Marital status (*n* = 1,950)					**0.009**
Single	104 (5.3)	86 (5.0)	10 (5.9)	8 (14.3)	
Married/de-facto relationship	1,846 (94.7)	1,639 (95.0)	159 (94.1)	48 (85.7)	
Smoking during pregnancy (*n* = 1,572)					0.73
No	1,166 (74.2)	1,050 (74.5)	87 (71.3)	29 (72.5)	
Yes	406 (25.8)	360 (25.5)	35 (28.7)	11 (27.5)	
Alcohol in pregnancy (*n* = 1,572)					0.55
No	294 (18.7)	266 (18.9)	19 (15.6)	9 (22.5)	
Yes	1,278 (81.3)	1,144 (81.1)	103 (84.4)	31 (77.5)	
Average BMI at 15 weeks ± SD, kg/m^2^ (*n* = 1,572)	24.9 ± 4.1	24.8 ± 4.1	25.3 ± 4.5	27.7 ± 3.9	**<0.001**
Average weight change between 15 and 20 weeks ± SD, kg (*n* = 1,572)	2.8 ± 1.9	2.8 ± 1.9	2.9 ± 1.7	2.9 ± 2.1	0.82
GDM (*n* = 1,572)					**0.03**
No	1,536 (97.7)	1,382 (98.0)	117 (95.9)	37 (92.5)	
Yes	36 (2.3)	28 (2.0)	5 (4.1)	3 (7.5)	
Child sex (*n* = 2,172)					**<0.001**
Male	1,095 (50.4)	950 (48.8)	121 (71.6)	24 (42.9)	
Female	1,077 (49.6)	997 (51.2)	48 (28.4)	32 (57.1)	
Pre-term delivery (<37 weeks) (*n* = 2,172)					0.12
No	2,087 (96.1)	1,872 (96.2)	164 (97.0)	51 (91.1)	
Yes	85 (3.9)	75 (3.9)	5 (3.0)	5 (8.9)	

a *Based on one-way ANOVA (for continuous variables) or chi-square test (for categorical variables)*.

b *n (%); indicates column percentages*.

*Significant at p < 0.05 level*.

**Table 2:** Fit indices.

**No. of classes**	**Class proportions**	**Sample-size adjusted BIC**	**LMRT for *k-1* vs. *k* classes**	**BLRT for *k-1* vs. *k* classes**	**Entropy**
		**Value**	***P-*value**	***P-*value**	
1	n/a	33,011	n/a	n/a	n/a
2	C1: 2,115 (97.4%) C2: 57 (2.6%)	32,784	0.005	<0.001	0.84
3	C1: 169 (7.8%) C2: 1,947 (89.6%) C3: 56 (2.6%)	32,717	0.009	<0.001	0.69
4	C1: 1,895 (87.2%) C2: 44 (2.0%) C3: 205 (9.4%) C4: 28 (1.3%)	32,681	0.288	<0.001	0.71

### Determinants of BMI Trajectories in the First 5 Years

Sex and maternal age were associated with membership of class 2, the trajectory characterized by early rapid gain. Girls were less likely than boys to belong to this trajectory [Relative risk ratio (RRR) (95% Confidence Interval (CI)): 0.17 (0.06–0.49)], as were children of older mothers [RRR (95% CI): 0.88 (0.79–0.99) for a maternal age increase of 1 year]. Maternal BMI and cord blood IGF-2 were associated with membership of class 3, the rapid gain after 12 months group. Children of mothers with higher BMI were more likely to belong to this trajectory [RRR (95% CI): 2.27 (1.15–4.48) for an increase of 1 kg/m^2^ in maternal BMI at 15 weeks]. Further, an increase of 1SD of cord blood IGF-2 was found to be inversely associated with membership [RRR (95% CI): 0.40 (0.16–0.98)]. Table 3 provides a full outline of the multivariate multinomial logistic regression analysis.

**Table 3:** Multinomial logistic regression for trajectory membership (*n* = 402).

	**Class 2 RRR (95% CI) *p*-value**	**Class 3 RRR (95% CI) *p*-value**
Maternal age, y	0.88 (0.79-0.99)^a^ **0.04**	0.98 (0.84–1.15) 0.84
**Educational attainment** **(vs. no third level)**		
Any third level	1.15 (0.27–4.85) 0.85	1.06 (0.09–11.54) 0.96
**Income level per year (vs**. **<** €**43 k)**		
€43–63 k	0.56 (0.18–1.70) 0.31	3.74 (0.56–25.10) 0.18
€64k+	0.85 (0.30–2.42) 0.76	1.45 (0.21–10.25) 0.71
**Marital status (vs. single)**		
Married/de-facto relationship	0.92, 0.99	0.27 (0.01–5.03) 0.38
Smoking during pregnancy (vs. not smoking)	1.01 (0.40–2.57) 0.98	0.64 (0.11–3.78) 0.62
Alcohol in pregnancy (vs. no alcohol)	4.20 (0.91–19.40) 0.07	0.29 (0.06–1.28) 0.06
BMI at 15 weeks, kg/m^2^	1.04 (0.66–1.65) 0.85	2.27 (1.15–4.48) **0.018**
**Child sex (vs. male)**		
Female	0.17 (0.06–0.49) **0.001**	4.06 (0.92–17.93) 0.065
Cord blood leptin (per 1SD increase)	1.08 (0.71–1.68) 0.73	1.13 (0.60–2.13) 0.71
Cord blood IGF-1 (per 1SD increase)	1.09 (0.71–1.68) 0.70	0.53 (0.23–1.22) 0.14
Cord blood IGF-2 (per 1SD increase)	1.22 (0.84–1.79) 0.29	0.40 (0.16–0.98) **0.046**

a *Reference category: class 1. Significant at p < 0.05 level*.

## Discussion

We have identified three distinct BMI trajectories in the first 5 years of life: normal, rapid BMI gain in the first 6 months, and rapid gain after 12 months. The trajectories were found to be associated with several determinants identified during pregnancy and labor, including sex, maternal age, maternal BMI, and cord blood IGF-2.

The trajectory patterns observed in this study are consistent with those identified in a previously published systematic review. Similarly to previous studies, we identified a normal trajectory and trajectories characterized by rapid gain at different time points. We did not identify a consistently high or low trajectory, which have been identified by some studies in the past (22). When using BMI as a measure of growth in longitudinal studies; raw BMI values or BMI z-scores may be used. BMI z-scores are standardized and indicate a child's position relative to same-age, same-sex children (40). Thus, BMI z-score trajectories indicate how children's BMI relative to peers changes over time, while raw BMI growth trajectories describe children's BMI change over time. While both may be used, some researchers have recommended raw BMI over z-scores for use in longitudinal analyses, as the within-child variability over time depends on the child's level of adiposity and findings presented in BMI units are more interpretable (41, 42)

In our study, boys were found to be more likely to belong to class 2, the trajectory characterized by early rapid gain, while no differences were identified for class 3. Further research is warranted to explain the potential sex difference in early BMI trajectories.

Adiposity, as measured by BMI, increases during the first year of life and then decreases. The adiposity rebound is the second rise in adiposity, which occurs between 3 and 7 years of age in individual children. It corresponds to fat cells starting to increase in number after an earlier phase of increasing then decreasing in size. The age the rebound occurs has been shown to predict obesity in later life, with an earlier AR (usually before 5 years of age) being associated with an increased risk of obesity (43, 44) and the timing of AR having been shown to be related to features of BMI trajectories (45). In this study early AR may be identified in class 3, as children in this class had a higher BMI at 5 years compared to 2 years of age (Supplementary Table 2 and Figure 1), indicating an AR at some point between these two measurements. As measurements of child weight and length were not made between 2 and 5 years of age, a more nuanced analysis of this time period was therefore not possible and as there was no follow-up past 5 years the timing of AR could not be identified in class 1 and 2.

The concept of fetal programming originates in a hypothesis that exposure to certain environmental influences during critical periods of development and growth may have significant consequences on an individual's long-term health. Developed by David Barker; it was originally called the Barker Hypothesis, then known as the Fetal Origin of Adult Disease, and now the Developmental Origins of Health and Disease (DOHaD) (46). According to this hypothesis, the fetus responds to a hostile uterine environment by developing adaptations that not only foster its immediate viability, but also its survival if a similar environment is encountered later in life (47). In terms of obesity development, there is a growing body of evidence that suggests that the origins of obesity and metabolic dysfunction can be traced back to the developing fetus responding to suboptimal conditions during critical periods of cellular proliferation, differentiation, and maturation by producing structural and functional changes in cells, tissues and organ systems, thus increasing the offspring's risk of developing a range of complex disorders, including obesity and metabolic dysfunction (48–50). In addition to fetal programming, the risk of childhood obesity and metabolic dysfunction may be further increased by a multitude of life course exposures, including SES, food production and marketing, and obesogenic environments. The effect of the exposure of these complex factors may be intergenerational; thus if mothers were exposed, contributing to their own obesity development, then their children are at higher of being exposed to all or some of the same factors (51, 52). High maternal pre-pregnancy BMI has previously been identified as the most frequently identified risk factor for membership of a rapid gain trajectory (22). In this study, BMI at 15 weeks gestation was associated with the trajectory characterized by early stable-high BMI followed by later rapid gain. How maternal BMI influences the BMI trajectory of the offspring is still not well-understood, however previous studies have found that over-nutrition *in utero* may lead to high-risk BMI trajectories during early childhood, and that this may occur through increased transfer of maternal energy substrates, such as glucose, lipids and amino acids to the fetus (53). Additionally, mothers with overweight or obesity may be more likely to experience placental dysfunction (54) and may be at higher risk of micronutrient deficiency (55). In this study we did not identify any associations between BMI trajectories or socioeconomic status, including maternal education, income, or marital status.

To our knowledge, this is the first study to examine latent BMI trajectories in childhood in association with cord blood leptin, IGF-1 and -2 levels. While no associations were identified for leptin or IGF-1, a higher IGF-2 concentration in the cord blood was found to be negatively associated with class 3, i.e., those experiencing rapid gain between 1 and 5 years. IGF-2 has been previously linked to intrauterine programming predisposing to cardiovascular risk in postnatal life (56). The “Dutch hunger winter” studies of the period of famine induced by the German-imposed food embargo in the western part of The Netherlands toward the end of World War II in the winter of 1944–1945 has provided support for the DOHaD concept, due to its unique nature. Although the food embargo was immediately lifted after liberation in May 1945, and children exposed to famine *in utero* during the hunger winter were well-nourished in childhood and had accelerated weight gain, decades later, they still experienced a higher incidence of cardiovascular disease (57). A cohort of individuals prenatally exposed to the Dutch Hunger Winter were tested six decades later, with results showing that the periconceptional exposure to famine was associated with reduced DNA methylation of the imprinted IGF-2 gene (58). The degree of IGF-2 methylation at birth has previously been linked to the development of childhood overweight and obesity (59). Cord blood IGF-2 concentrations have previously been found to be related to IGF-2 levels at age five, which in turn have been related to fat mass (24), however the relationship between cord blood IGF and growth in childhood remains largely unexplored and further research is warranted.

### Strengths and Limitations

This study has several strengths. The large sample size and prospective design allowed the collection of repeated weight and height measurements and the evaluation of longitudinal childhood BMI trajectories. All weight and height data were obtained by trained staff using standardized instruments using strict Standard Operating Procedures. As the sample was recruited from the only maternity hospital in Cork, Ireland it included mothers and children from a broad range of social circumstances. Mean maternal age (30.9 years) was comparable with that reported nationally for the same year (31.5) (60) and the high prevalence of Caucasian women (98%) mirrors the Irish Census of 2006 (95%) (61). Income was normally distributed across the categories, with 45% of the participants reporting household incomes between 43,000 and 84,000 per year, and the average household income in Ireland in 2011 53,000 per year.

However, limitations remain. The comparative strength and limitations of GMM over other alternative approaches has been the subject of some debate (62). A more straightforward and commonly used method is following a child's BMI or z-score over time, with excessive growth indicated by crossing major percentile lines on a standard growth chart (63). Compared to the relatively complex and computer-intensive nature of GMM, this method is simple to implement. Furthermore, the assignment of children to a distinct developmental pattern is based on their highest estimated group-membership probability to the identified pattern. Thus, these latent patterns should not be considered as the actual developmental patterns but, rather, as approximations of more complex ones. Consequently, the findings will reflect associations of determinants with a model-derived class variable based on modeled BMI patterns. While GMM approaches remove the constraint on within-class variation in the LCGA method, disadvantages with such approaches have been highlighted with respect to interpretability and the identification of non-existent subclasses (62). However, while choosing the correct model and number of classes in GMM is not straightforward, the crossing of percentiles method assumes growth to be a linear function of size at different ages. Conversely, GMM is capable of modeling non-linear growth curves, estimating individual trajectories and identifying distinctive subgroups in the population, and we therefore believe this method provides an important dimension for consideration and that these advantages may outweigh its complexity.

Lastly, in terms of the study sample and data some limitations must be noted. Firstly, some variables of interest could not be included due to the lack of availability, including parity and gestational weight gain. While the study sample was comparable to the general population in several aspects as detailed above, the women participating in the study were better educated than the general population, with 89% having obtained post-secondary school education, compared to 45% in the 2006 census (61). Further, the issue of missing data must be acknowledged. Of the 2,172 children with data collected after birth, 1,135 (52%) were followed up at 5 years. However, as FIML was used to account for missing data, all children were included in the trajectory analysis. Sensitivity analyses using subjects with no missing BMI data in the first 5 years (*n* = 915) showed similar trajectory patterns, thus illustrating the robustness of the extracted BMI trajectories. For the multinomial regression analysis, only 402 children were included in the final model. The low number is a result of leptin only being collected in a subset of children (*n* = 405), and the missing data may therefore be deemed missing at random. This may be further demonstrated in Supplementary Table 3, which shows population characteristics for participants with and without leptin data. Further research with larger numbers of children is required.

## Conclusion

We identified three distinct BMI trajectories in the first 5 years of life which were associated with maternal age and BMI, sex, and cord blood concentration of IGF-2. The potential public health and clinical implications of these findings are important. First, identification of BMI trajectories in early childhood may be helpful in identifying high-risk groups. Second, the assessment of prenatal determinants of BMI trajectories may enhance the understanding of etiologic pathways of childhood obesity and potentially target interventions. Further research and extended follow-up to examine the effects of early childhood growth patterns are required to understand this complex public health issue.

## Data Availability Statement

The datasets presented in this article are not readily available and access to the data must be applied for through the principal investigators. Requests to access the datasets should be directed to d.murray@ucc.ie.

## Ethics Statement

The studies involving human participants were reviewed and approved by Clinical Research Ethics Committee of the Cork Teaching Hospitals. Written informed consent to participate in this study was provided by the participants' legal guardian/next of kin.

## Author Contributions

MM was involved in the study conception and design, requested the data sets, performed statistical analysis of the data, and drafted the manuscript. RB and DM were involved in the study conception and design and critically reviewed the manuscript. FB was involved in the study conception and design, critically reviewed the manuscript, and provided statistical support. FM and CH critically reviewed the draft manuscript. MK and CN analyzed and supplied the leptin data. All authors approved the final manuscript as submitted and agree to be accountable for all aspects of the work.

## Conflict of Interest

The authors declare that the research was conducted in the absence of any commercial or financial relationships that could be construed as a potential conflict of interest.
