# Supplementary material for: Body Mass Index Trajectories in the First 5 Years and Associated Antenatal Factors
Source: Front Pediatr. 2021 Feb 19;9:622381. doi: 10.3389/fped.2021.622381 (PMC7933027; doi:10.3389/fped.2021.622381)
Supplement: Supplementary file 1 [file Table_1.docx]

**Supplementary table 1. Available data at each time point according to BMI trajectory**

|  | **All**  **(n=2,172)** | **Class 1**  **(n=1,947)** | **Class 2**  **(n=169)** | **Class 3**  **(n=56)** |
| --- | --- | --- | --- | --- |
| **Birth** | n=2,137/98.4%^a^ | n=1,916/98.4% | n=166/98.2% | n=55/98.2% |
| **2 months** | n=1,807/83.2% | n=1,595/81.9% | n=162/95.9% | n=50/89.3% |
| **6 months** | n=1,723/79.3% | n=1,504/77.2% | n=164/97.0% | n=55/98.2% |
| **12 months** | n=1,538/70.8% | n=1,352/69.4% | n=135/80.0% | n=51/91.1% |
| **2 years** | n=1,432/66.0% | n=1,255/64.5% | n=127/75.1% | n=52/92.9% |
| **5 years** | n=1,135/52.3% | n=985/50.6% | n=96/56.8% | n=54/96.4% |

^a^Number and proportion of sample with available data
