# Supplementary material for: Body Mass Index Trajectories in the First 5 Years and Associated Antenatal Factors
Source: Front Pediatr. 2021 Feb 19;9:622381. doi: 10.3389/fped.2021.622381 (PMC7933027; doi:10.3389/fped.2021.622381)
Supplement: Supplementary file 2 [file Table_2.DOCX]

**Supplementary table 2. BMI ±SD at each time point according to BMI trajectory**

|  | **All** | | **Class 1** | | **Class 2** | | **Class 3** | |
| --- | --- | --- | --- | --- | --- | --- | --- | --- |
|  | **Boys** | **Girls** | **Boys** | **Girls** | **Boys** | **Girls** | **Boys** | **Girls** |
| **Birth** | 13.67  ±1.43 | 13.66  ±1.43 | 13.56  ±1.38 | 13.61  ±1.39 | 14.52  ±1.56 | 14.59  ±1.78 | 13.61  ±1.32 | 13.75  ±1.84 |
| **2 months** | 16.29  ±1.41 | 15.65  ±1.27 | 16.02  ±1.28 | 15.53  ±1.18 | 17.92  ±1.06 | 17.50  ±1.16 | 17.23  ±1.19 | 16.32  ±130 |
| **6 months** | 17.63  ±1.53 | 17.15  ±1.38 | 17.22  ±1.20 | 16.93  ±1.17 | 20.07  ±0.99 | 19.94  ±1.10 | 18.70  ±1.50 | 18.06  ±1.28 |
| **12 months** | 17.44  ±1.40 | 17.02  ±1.38 | 17.12  ±1.18 | 16.85  ±1.25 | 19.35  ±0.98 | 19.52  ±1.18 | 18.53  ±1.91 | 17.78  ±1.19 |
| **2 years** | 16.91  ±1.28 | 16.65  ±1.30 | 16.71  ±1.18 | 16.53  ±1.20 | 17.91  ±1.04 | 17.93  ±1.51 | 18.31  ±1.96 | 17.71  ±1.65 |
| **5 years** | 16.12  ±1.36 | 16.03  ±1.45 | 15.83  ±1.01 | 15.76  ±1.16 | 16.91  ±0.94 | 17.20  ±1.25 | 20.06  ±1.66 | 19.44  ±0.99 |
