# Supplementary material for: Body Mass Index Trajectories in the First 5 Years and Associated Antenatal Factors
Source: Front Pediatr. 2021 Feb 19;9:622381. doi: 10.3389/fped.2021.622381 (PMC7933027; doi:10.3389/fped.2021.622381)
Supplement: Supplementary file 3 [file Table_3.DOCX]

**Supplementary table 3. Distribution of population characteristics for participants with and without leptin data**

|  | **All participants** | **Participants with leptin data** | **Participants with missing leptin data** | **P-value^a^** |
| --- | --- | --- | --- | --- |
| **Average age**  ±**standard deviation (SD), years** (n=1,572) | 30.0 ±4.4 | 30.3 ±4.2 | 30.0±4.5 | 0.23 |
|  | **%**^b^ | **%** | **%** |  |
| **Ethnicity** (n=1,950) |  |  |  | 0.60 |
| Irish or other Caucasian | 98.0 | 98.2 | 97.5 |  |
| Other | 2.0 | 1.8 | 2.5 |  |
| **Educational attainment** (n=1,949) |  |  |  | 0.57 |
| No college/university education | 10.7^b^ | 9.8 | 10.9 |  |
| Any college/university education | 89.3 | 90.2 | 89.2 |  |
| **Income level per year** (n=1,873) |  |  |  | 0.61 |
| < €43k | 27.5 | 27.8 | 27.1 |  |
| €43-63k | 23.3 | 22.5 | 25.4 |  |
| €64+ | 49.2 | 49.5 | 47.5 |  |
| **Marital status** (n=1,950) |  |  |  | 0.91 |
| Single | 5.3 | 5.2 | 5.4 |  |
| Married/de-facto relationship | 94.7 | 94.8 | 94.6 |  |
| **Smoking during pregnancy** (n=1,572) |  |  |  | 0.85 |
| No | 74.2 | 74.6 | 74.1 |  |
| Yes | 25.8 | 25.4 | 25.9 |  |
| **Alcohol in pregnancy** (n=1,572) |  |  |  | 0.67 |
| No | 18.7 | 17.9 | 18.9 |  |
| Yes | 81.3 | 82.1 | 81.1 |  |
| **Average BMI at 15 weeks** ±**SD, kg/m^2^** (n=1,572) | 24.9 ±4.1 | 25.1±3.9 | 24.8±4.2 | 0.14 |
| **Average weight change between 15 and 20 weeks** ±**SD, kg** (n=1,572) | 2.8 ±1.9 | 2.6±1.4 | 2.9±1.7 | 0.60 |
| **GDM** (n=1,572) |  |  |  | 0.23 |
| No | 97.7 | 98.0 | 95.9 |  |
| Yes | 2.3 | 2.0 | 4.1 |  |
| **Child sex** (n=2,172) |  |  |  | 0.51 |
| Male | 1,095 (50.4) | 52.0 | 50.1 |  |
| Female | 1,077 (49.6) | 48.0 | 49.9 |  |
| **Preterm delivery(<37 weeks)** (n=2,172) |  |  |  | 0.64 |
| No | 96.1 | 96.5 | 96.0 |  |
| Yes | 3.9 | 3.5 | 4.0 |  |

^a^ Based on one-way ANOVA (for continuous variables) or chi-square test (for categorical variables).

^b^ n (%); indicates column percentages.
