# Supplementary material for: Body Mass Index Trajectories in the First 5 Years and Associated Antenatal Factors
Source: Front Pediatr. 2021 Feb 19;9:622381. doi: 10.3389/fped.2021.622381 (PMC7933027; doi:10.3389/fped.2021.622381)
Supplement: Supplementary file 4 [file Data_Sheet_1.docx]

**Supplementary Figure 1. Flow chart for children included at each stage of the study**

SCOPE: 1,768 women recruited in early pregnancy

231 women did not continue with the study

BASELINE Stream 2:

646 infants recruited after delivery

BASELINE Stream 1: 1,537 SCOPE participants consented for their infants to participate

1,770 had observations missing on one or more of the variables included in the regression model. The large proportion of missing data was primarily due to leptin only being analysed in a subset of children (n=405)

402 children with no missing data were included in regression analysis

11 women initially consented but had no data collected after birth

2,172 children had data collected after birth and were included in GMM analysis
